# 4074 The Role of Immortal Time Bias When Linking Treatment to Outcomes among Older Patients with Incident Hodgkin Lymphoma (HL) using Surveillance, Epidemiology and End Results (SEER)-Medicare Data

**DOI:** 10.1017/cts.2020.151

**Published:** 2020-07-29

**Authors:** Angie Mae Rodday, Theresa Hahn, Peter K Lindenauer, Susan K Parsons

**Affiliations:** 1 Tufts University; 2 Roswell Park Comprehensive Cancer Center; 3 Baystate Health; 4 Tufts Medical Center

## Abstract

OBJECTIVES/GOALS: Older patients with HL have worse outcomes than younger patients, which may reflect treatment selection, including fewer chemotherapy cycles. Immortal time bias exists when patients must survival to initiation, and even completion, of treatment. We described treatment length and death to evaluate the extent of immortal time bias. METHODS/STUDY POPULATION: This retrospective cohort study utilized SEER-Medicare data from 1999-2014. Patients diagnosed with incident advanced stage HL at age ≥65 years and enrolled in Medicare Part A and B fee for service were included. Chemotherapy or radiotherapy treatment initiated within 4 months of diagnosis was determined from inpatient, outpatient, and physician/supplier claims. No treatment was defined by lack of treatment claims. Dates from claims were used to define length of treatment; ≥4 months of treatment indicated complete chemotherapy cycles. Date of death was obtained from Medicare data. Analyses were limited to 1 year post-diagnosis. Summary statistics were used to describe treatment length and subsequent death. RESULTS/ANTICIPATED RESULTS: We included 1492 advanced stage HL patients with a mean age of 76 years (SD = 7). 428 (29%) patients had no documented treatment; 397 (27%) were treated <4 months indicating fewer chemotherapy cycles; and 667 (45%) were treated for ≥4 months indicating complete chemotherapy cycles. Among those with no documented treatment, 15% died within 1 month of diagnosis with 78% dying by 1 year post-diagnosis. Among those treated <4 months, 36% died within 1 month of their last treatment claim with 64% dying by 1 year post-diagnosis. Among those treated ≥4 months, 7% died within 1 month of their last treatment claim with 14% dying by 1 year post-diagnosis. DISCUSSION/SIGNIFICANCE OF IMPACT: Few untreated patients died within 1 month of diagnosis. One-third of patients treated <4 months died soon after completion of treatment, while patients treated longer survived longer, suggesting some patients did not survive to complete treatment. To account for this immortal time bias, landmark analysis will be used to assess the relationship between treatment and survival.

